# Multilevel Understanding of the Impact of Individual- and School-Level Determinants on Lipid Profiles in Adolescents: The Cross-Level Interaction of Food Environment and Body Mass Index

**DOI:** 10.3390/nu14102068

**Published:** 2022-05-15

**Authors:** Wei-Ting Lin, Yu-Ting Chin, Pei-Wen Wu, Sharon Tsai, Meng-Hsueh Chen, Chiao-I Chang, Yu-Cheng Yang, Chun-Ying Lee, David W. Seal, Chien-Hung Lee

**Affiliations:** 1 Department of Social, Behavioral, and Population Sciences, School of Public Health and Tropical Medicine, Tulane University, New Orleans, LA 70112, USA; wtlin0123@gmail.com (W.-T.L.); dseal@tulane.edu (D.W.S.); 2 Department of Public Health, College of Health Sciences, Kaohsiung Medical University, Kaohsiung 80708, Taiwan; kiki13336586@gmail.com (Y.-T.C.); catstar1211@gmail.com (P.-W.W.); cher7149@gmail.com (M.-H.C.); s370002000@yahoo.com.tw (C.-I.C.); l19920929@gmail.com (Y.-C.Y.); 3 Department of Laboratory Medicine, Kaohsiung Municipal Siaogang Hospital, Kaohsiung 81267, Taiwan; 870718@kmuh.org.tw; 4 Jianan Psychiatric Center, Ministry of Health and Welfare, Tainan 71742, Taiwan; 5 Department of Family Medicine, Kaohsiung Medical University Hospital, Kaohsiung Medical University, Kaohsiung 80756, Taiwan; cying@ms19.hinet.net; 6 Research Center for Environmental Medicine, Kaohsiung Medical University, Kaohsiung 80708, Taiwan; 7 Department of Medical Research, Kaohsiung Medical University Hospital, Kaohsiung Medical University, Kaohsiung 80756, Taiwan; 8 Office of Institutional Research & Planning, Secretariat, Kaohsiung Medical University, Kaohsiung 80708, Taiwan

**Keywords:** lipid profile, sugar-sweetened beverage, physical activity, body mass index, food environment near the school, cross-level influence, multilevel effect, adolescents

## Abstract

Adolescents with comparable personal risk factors may have different lipid profiles because of the school’s context. Lipid determinants in adolescents should be considered using a multilevel perspective. This multilevel study investigated the effects of individual-level and school-level factors on lipid profiles in adolescents and evaluated the cross-level influence of lipid determinants. A representative adolescent cohort (*n* = 2727) was randomly selected from 36 schools in three diverse economic areas in Taiwan and assessed for their personal dietary patterns, physical parameters, and lipid profiles. For individual-level factors, both low physical activity and high body mass index (BMI) were associated with elevated triglyceride (TG), low-density lipoprotein cholesterol (LDL-C), and total cholesterol (TC) levels, and a sugar-sweetened beverage intake of >500 mL/day was associated with increases of 5.97 and 6.12 mg/dL in LDL-C and TC levels, respectively, compared with abstinence. Regarding school-level factors, students in schools with ≥2 health promotion programs per year had a 5.27 mg/dL lower level of LDL-C than those in schools with 0–1 program, and students in schools with ≥46 food outlets within 600 m of the school had 6.90 and 13.3 mg/dL higher levels of TG and TC, respectively, than those in schools with <46 food outlets. School context modified the individual-level positive correlation between BMI and TG level (the *p*-value for the random-slope effect was 0.003). In conclusion, individual-level and school-level factors exert a multilevel effect on adolescent lipid profiles. The food environment near the school has a stronger cross-level impact on individual TG levels in adolescents with a high BMI than in those with a normal BMI.

## 1. Introduction

An adverse lipid profile, including high triglyceride (TG), low high-density lipoprotein cholesterol (HDL-C), elevated low-density lipoprotein cholesterol (LDL-C), and increased total cholesterol (TC) levels, is closely associated with multiple health risks, such as arteriosclerosis, metabolic syndrome, type-2 diabetes, and cardiovascular disease [1]. The concomitant clinical treatment of these diseases considerably affects the health care system [2,3]. Epidemiological investigations have demonstrated that an undesirable lipid profile that develops during adolescence may persist into adulthood [4,5]. Adolescents with high levels of blood lipids have also been observed to have a high risk of developing cardiometabolic disease in adulthood [5,6,7]. Therefore, the lipid profiles of adolescents merit greater attention.

People with comparable personal characteristics may have diverse levels of health experiences, depending on where they live, because of the differences in environment, culture, policy, economy, and geography [8,9,10]. In adults, studies have revealed that dyslipidemia is a consequence of complex interactions among individual and environmental variables; that is, in addition to personal genetic predisposition and unhealthy lifestyles, outdoor built environments also exert a cross-level effect on the development of abnormal lipid levels [1,2,11,12]. A meta-analysis of 103 observational and 40 interventional studies reported that elevated greenspace exposure was beneficial for preventing a broad range of health conditions, including dyslipidemia, coronary heart disease, and stroke [12]. In adolescents, poor individual dietary intake and low physical activity have been linked to an increased risk of dyslipidemia [13,14,15]. Nevertheless, fast-food restaurants are often concentrated within a short walking distance of schools, exposing schoolchildren to poor-quality food [16]. School-level factors, such as food supply density near schools and health promotion programs conducted in schools, may also have an effect on adolescent lipid profiles. Efforts to identify and analyze individual-level and school-level factors that influence lipid levels in schoolchildren can support development strategies to improve adolescent lipid health and reduce the number of negative consequences in adulthood.

Adolescents may have homogeneous and clustered lipid profiles within the same schools and exhibit heterogeneous lipid levels between schools; however, the correlation of school-related contextual factors and student-related individual factors with intraschool clustering and interschool heterogeneity in lipid profiles remains to be determined. Another question is whether the school’s context has a modifying effect on the relationship between individual factors and lipid levels. One multi-country, multilevel study indicated that country context can modify the association between body mass index (BMI) and systolic blood pressure (SBP); that is, BMI has a stronger influence on SBP in certain countries than in others [17]. Such data can be used to develop school-level prevention programs for adolescent lipid health.

In a 2010–2011 nationwide study in Taiwan conducted to monitor the nutritional conditions and health of children, the prevalence of central obesity, high TG, and low HDL-C for adolescents aged 12–18 years were reported as 17.7–18.1%, 3.0%, and 12.3–19.3%, respectively [15]. One longitudinal study conducted in southern Taiwan reported that adolescents with an initial increased level of triglycerides had a 5.7-fold risk for persistent abnormality after 2.2 years, as compared with those with a normal status at baseline [18]. The distribution and determinants of blood lipids among adolescents should be considered using a multilevel perspective.

This study proposed three hypotheses. First, school context might have an effect on lipid profiles in adolescents. Second, specific individual-level and school-level factors might contribute to the difference in blood lipid levels between adolescents. Third, individual-level and school-level factors might have a cross-level influence on lipid profiles in adolescents. To elucidate the proposed hypotheses, this multilevel study examined the following: (1) the extent to which differences in lipid levels in adolescents are attributable to school context; (2) the effects of individual-level and school-level factors on adolescent blood lipid levels; and (3) the degree to which individual-level and school-level factors have a cross-level influence on lipid levels.

## 2. Materials and Methods

### 2.1. Participants

**Figure 1:**
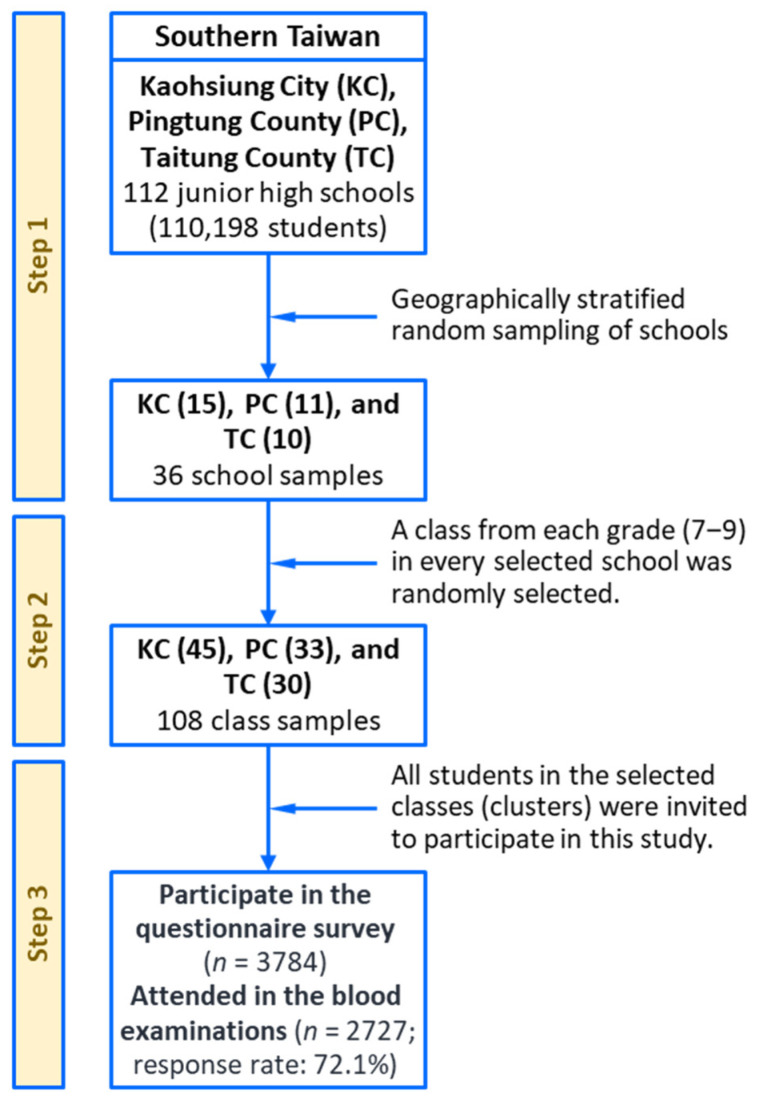
A multistage, geographically stratified cluster-sampling scheme for the southern Taiwan multilevel study.

We conducted this multilevel study on risk profiles for adolescent metabolic syndrome from 2007 to 2009. This study was a large-scale, multiarea, cross-sectional investigation designed to monitor and assess the risk factors and behaviors affecting adolescent cardiometabolic health in southern Taiwan. The research population comprised teenagers aged 12–16 years who were enrolled in junior high schools in Kaohsiung City, Pingtung County, and Taitung County (Taiwan), which are three areas with different levels of urbanization. The details of the research procedures are presented in Figure 1 and have been reported elsewhere [19,20,21,22]. We used a multistage, geographically stratified cluster-sampling method to recruit a representative sample of adolescents. A total of 36 schools were randomly selected from the study areas, and three classes, representing grades 7–9, were randomly selected from every school. For the selected schools, a class was considered to be a cluster (approximately 28–33 students), and all the students in the selected classes were invited to participate in this research. A total of 2727 adolescents (1328 boys and 1399 girls, response rate: 72.1%) answered a questionnaire and participated in anthropometric surveys and clinical blood examinations. This research protocol was reviewed and approved by the Institutional Review Board of Kaohsiung Medical University Hospital (KMUHIRB-20120103). All data collection was conducted in accordance with the guidelines for ethical conduct in human research. Written informed assent and consent were obtained from the adolescents and their parents or guardians, respectively.

### 2.2. Individual-Level Factors

A structured questionnaire for investigating individual-level risks was used to collect data on demographics, dietary intake, physical activity, lifestyle behavior, and personal disease history [19,20,21,22]. The questionnaire contains a wide range of items but does not particularly focus on the problems of blood lipids. With the management and assistance of teachers, two research assistants specifically trained for this study collected information and anthropometric measurements from the participants. Demographic information included age, sex, ethnicity, and school and home locations. We assessed individual dietary intake over the one-month period prior to the study by using a verified semiquantitative food-frequency questionnaire with the following categories: milk, dairy products, eggs, meats, fresh fruits, fried foods, and sugar-sweetened beverages (SSB) [19,23]. Participants who consumed one or more servings of any category of SSBs per week during the prior month were defined as SSB drinkers [20]. The daily consumption of various SSBs, including soft drinks, sweetened teas, fruit drinks, and sports drinks, were added up and were classified as non-intake or an intake of 1–350, 351–500, or >500 mL/day [24]. Nutritional data obtained from the Taiwanese Food and Nutrients Databank were used to quantify daily caloric intake using individual food intakes [25]. In terms of physical activity, the minutes per day and the number of days per week spent on each activity by each student on weekdays and weekends during the month preceding the study were converted to metabolic equivalent task-minutes (MET-min) [19]. The MET levels of each activity were estimated using an energy expenditure guideline developed for youths [26]. We categorized adolescents into three groups according to tertiles of the overall MET-min. Anthropometric data, including weight and height, were physically measured by qualified researchers using standardized procedures. BMI, which was calculated using the ratio of weight (kg) to height (m^2^), was used to characterize body weight.

### 2.3. School-Level Factors

We used another questionnaire for investigating the effect of health promotion programs on lipid profiles. All types of health promotion programs (weight control, healthy dietary intake, tooth brushing methods and oral hygiene education, and smoking prevention and cessation) implemented by each school within the 2 years prior to the study were recorded. On the basis of the assumption that an adolescent can walk 600 m in 10 min, we utilized Google Maps (Google Inc., Mountain View, CA, USA) to create a 600-m-radius zone around each school and traveled to each zone to count the number of food outlets and convenience stores within each area [27]. The investigated food outlets included fast-food restaurants, beverage shops, and eateries (comprising roadside stalls, noodle bars, and snack bars).

### 2.4. Lipid Measurements

After the collection of written informed assents and consents from the participants and their parents or guardians, researchers rechecked and confirmed the participant list and talked with the school representatives to establish an appropriate date when blood samples could be collected from the participants. Usually, the work of blood specimen collection was carried out 3 weeks after the questionnaire survey. We obtained the clinical samples in the morning through venipuncture at each school’s health center, after participants fasted for 10 h. The lipid profiles of serum TG, HDL-C, LDL-C, and TC levels (mg/dL) were enzymatically quantified using an autoanalyzer with commercially available reagents (TBA-c16000 automatic analyzer, Toshiba, Tokyo, Japan) [28].

### 2.5. Statistical Analysis

Four statistical procedures were used to analyze the variability of the four lipid levels. First, sex, ethnicity, age, and daily caloric intake were considered as potential confounding factors, and they were controlled for in all multivariate analyses [29,30]. Second, we plotted a series of caterpillar plots, using ordered means and 95% confidence intervals, to illustrate the distribution of lipid levels across schools. A random-intercept mixed-effects model with no explanatory variables was used for the four lipid levels, to examine possible school-level random effects [8]. A significant random effect indicated a notable school-context effect. Third, the effects of individual-level and school-level factors on the four lipid levels were assessed using multiple linear regression models. Fourth, each lipid level was analyzed using a multilevel (mixed-effects) linear regression model (MLRM), with adolescents at the first level and schools at the second level [8,9,10]. Three MLRMs were used to evaluate the multilevel effects of the study factors [17]. Model I illustrated the variability in lipid levels among individuals and among schools (measured as interindividual and interschool variances, respectively); therefore, this did not include any explanatory variables. Model II assessed the association between individual-level factors and serum lipids. Model III evaluated the multilevel effects of individual-level and school-level factors on lipid levels by additionally including the school-level factors in Model II. We compared the two nested models using likelihood-ratio tests, to examine the significance of random-intercept and random-slope effects in the MLRMs.

In Models I to III, the individual-level variance ($V_{ind}$) and school-level variance ($V_{sch}$) for random effects were computed; these values were then used to calculate the individual-level (${\mathrm{PCV}}_{ind}$) and school-level (${\mathrm{PCV}}_{sch}$) proportional changes of variance (PCV) in lipid levels, as explained by individual-level or school-level factors, respectively [8]. $V_{ind}$ and $V_{sch}$ were also used to calculate the variance partition coefficient (VPC) [9,17]. We designated $M_{0}$ and $M_{1}$ as the reference and study models, respectively, and calculated PCV and VPC using the following formulas [8,9,17]:

(1) $${\mathrm{PCV}}_{ind}=\frac{V_{ind}\left(M_{0}\right)-V_{ind}\left(M_{1}\right)}{V_{ind}\left(M_{0}\right)}$$

(2) $${\mathrm{PCV}}_{sch}=\frac{V_{sch}\left(M_{0}\right)-V_{sch}\left(M_{1}\right)}{V_{sch}\left(M_{0}\right)}$$

(3) $$\mathrm{VPC}=\frac{V_{sch}}{V_{sch}+V_{ind}}$$

VPC measures the general clustering (or similarity) of individual lipid levels in a school; in its simplest form (e.g., Model I), it corresponds to the intraclass correlation [17]. Because school context had a significant random-coefficient effect on the association between BMI and TG level, we centered BMI by subtracting each BMI value from the mean BMI (21.3 kg/m^2^) and used an unstructured covariance matrix to calculate the variances and covariances for random-intercept and random-slope effects. In Models II and III for TG level, the $V_{sch}$ was a function of BMI; thus, VPC was also a function of BMI [9]. We plotted the VPC curves to interpret the modifying effect of school context on the individual-level relationship between BMI and TG levels.

## 3. Results

**Figure 2:**
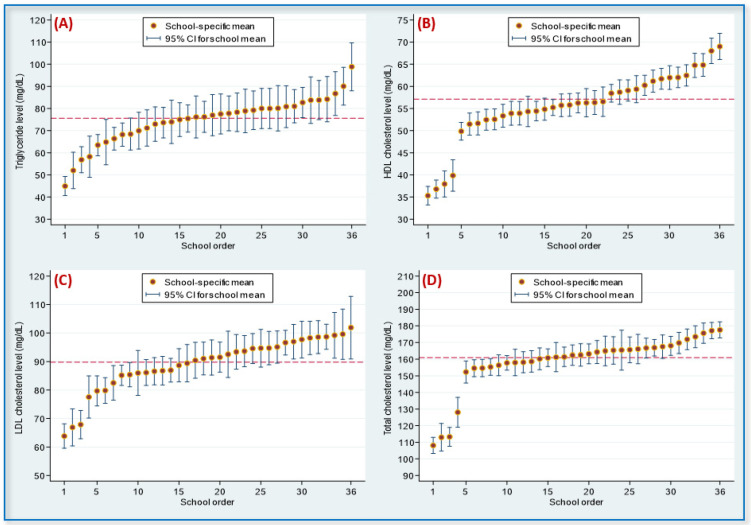
Caterpillar plots for the distribution of serum lipids among adolescents in 36 studied schools: (**A**) triglyceride; (**B**) high-density lipoprotein (HDL) cholesterol; (**C**) low-density lipoprotein (LDL) cholesterol; (**D**) total cholesterol. Note. School-level random-intercept effects for the 4 lipids were significant (all *p*-values were < 0.001). The dashed line represented the overall mean: 75.6 mg/dL for triglyceride, 57.1 mg/dL for HDL cholesterol, 89.8 mg/dL for LDL cholesterol, and 161.0 mg/dL for total cholesterol. CI, confidence interval.

Figure 2 presents four caterpillar plots for the distributions of serum TG, HDL-C, LDL-C, and TC levels among adolescents from the 36 schools. Likelihood-ratio tests indicated that all school-level random-intercept effects for the four lipid levels were significant (all values were at *p* < 0.001).

Table 1 displays the association of demographics and daily caloric intake with lipid profiles in adolescents. Girls had higher levels of HDL-C, LDL-C, and TC than boys did, and students of aboriginal origin had lower levels of all four lipids than students of Fukienese origin. Age was negatively correlated with TC level. No significant association was found between daily caloric intake and the four lipid levels.

Table 2 lists the adjusted effects of individual-level factors on the four lipid profiles. Milk intake was associated with higher levels of LDL-C and TC, whereas dairy product intake was correlated with elevated HDL-C levels. Adolescents with high SSB consumption (>500 mL/day) had LDL-C and TC levels that were 6.83 mg/dL and 4.52 mg/dL higher, respectively, than those of adolescents who abstained from drinking SSBs. Compared with adolescents with high levels of physical activity (≥2140.5 MET-min/week), participants with low physical activity levels (<952.5 MET-min/week) had a 5.52 mg/dL higher TG level, a 3.67 mg/dL higher LDL-C level, and a 1.54 mg/dL lower HDL-C level. BMI was positively correlated with TG, LDL-C, and TC levels and was negatively correlated with the HDL-C level.

Table 3 presents the adjusted effects of school-level factors on lipid profiles. Adolescents whose schools conducted ≥2 health promotion programs per year had HDL-C, LDL-C, and TC levels that were 4.53, 6.02, and 9.68 mg/dL lower, respectively, than those of adolescents whose schools conducted 0–1 program per year. Adolescents studying in schools with ≥46 food outlets within 600 m had higher levels of the four lipids than those studying in schools with <46 food outlets. A high number of fast-food restaurants, beverage shops, and eateries near the schools was associated with high HDL-C, LDL-C, and TC levels in the participants. The relationship between the number of convenience stores and the four lipid levels was inconsistent; it was positively correlated with HDL-C levels, negatively correlated with LDL-C levels, and had no correlation with TG and TC levels.

**Figure 3:**
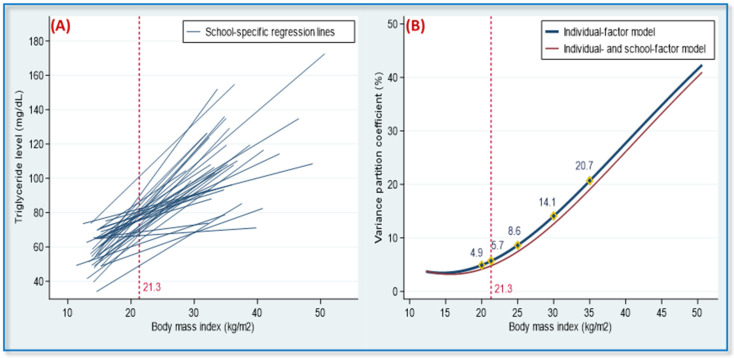
School-specific regression lines and variance partition coefficients (VPC) in 36 adolescent populations. (**A**) The heterogeneity of the regression slopes for the association between body mass index (BMI) and triglyceride levels. (**B**) The VPC revealed a function of BMI values. Note: VPC denotes the percentage of the total variance in terms of individual triglyceride levels, which was attributed to between-schools variability. In the individual-factor model, the VPC values were 4.9, 5.7, 8.6, 14.1, and 21.7% for adolescents with a BMI of 20, 21.3 (mean), 25, 30, and 35, respectively, and in the individual- and school-factor model, the corresponding VPC values were 4.1, 4.8, 7.4, 12.6, and 19.0%. VPC values were adjusted for sex, age, ethnicity, total daily calorie intake, and physical activity. The overall mean of BMI for the 36 adolescent populations was 21.3.

Table 4 displays the multilevel effects of individual-level and school-level factors on TG level. Lower physical activity and higher BMI were associated with higher TG levels; these two factors explained 8.1% of the interschool variance and 11.3% of the interindividual variance in TG levels, respectively (Model II). The positive correlation between BMI and TG varied according to school (Figure 3A), with schools significantly modifying the relationship between the two variables (variance of BMI–TG slopes between schools, 0.64, *p* < 0.05, Model II). The VPC versus BMI curve was J-shaped in both Models II and III (Figure 3B). Among school-level factors, the presence of ≥46 food outlets within 600 m of a school was associated with an increase of 6.90 mg/dL in the participants’ TG level.

Table 5 lists the multilevel effects of individual-level and school-level factors on HDL-C level. Adolescents who consumed ≥1 serving/week of dairy products had an HDL-C level that was 0.94 mg/dL higher than that of adolescents who consumed <1 serving/week of dairy products (Model II). BMI was inversely associated with HDL-C levels, even when we controlled for school-level factors (Models II and III). Having ≥2 health promotion programs per year was associated with a decrease of 4.53 mg/dL in HDL-C levels, whereas the presence of ≥46 food outlets within 600 m of a school was associated with an increase of 5.98–9.91 mg/dL in HDL-C levels (Model III). These two factors accounted for 54.1% of the interschool variance.

Table 6 presents the multilevel effects of individual-level and school-level factors on LDL-C and TC levels. High milk intake, elevated SSB consumption, low physical activity, and increased BMI were all associated with higher levels of LDL-C and TC (Model II). These four individual factors accounted for 8.9 and 2.1% of the interschool variance in LDL-C and TC levels, respectively, and 5.8 and 1.9% of the interindividual variance. For the school-level factors, having ≥2 health promotion programs per year was associated with a decrease of 5.27 mg/dL in LDL-C level; in contrast, the presence of ≥46 food outlets within 600 m of a school was associated with an increase of 13.2–13.3 mg/dL in TC level (Model III). The two school-level factors explained 18.9% (Model II vs. Model III, 8.9 → 27.8%) and 32.1% (Model II vs. Model III, 2.1→ 34.2%) of the interschool variance in LDL-C and TC levels, respectively.

## 4. Discussion

This study examined the effects of individual-level and school-level factors on four lipid levels in adolescents. In general, physical activity, dairy intake, SSB consumption, and BMI had fixed effects on lipid levels, whereas health promotion programs and the density of food outlets near schools had school-level influences on specific lipid levels. Moreover, school context had a cross-level modifying effect on the individual-level association between BMI and TG levels in adolescents.

For risk factors at the individual level, our investigation revealed that a high TG level in adolescents was associated with high BMI and low physical activity (TG levels increased by 2.59 mg/dL per unit increase in BMI; TG levels increased by 5.25 mg/dL for physical activity <952.5 vs. ≥2140.5 MET-min/week (Table 4, Model III)). These two factors explained 11.4% of the interindividual variance in the multilevel structure. Studies on adolescents in which dual-energy X-ray absorptiometry was used to measure fat and muscle mass have reported that high TG levels are associated with higher body-fat mass but not exercise-related isolated muscle mass [31,32]. These findings suggest that the association between high levels of physical activity and a low TG level may be related to a decrease in body fat instead of an increase in muscle mass.

People with similar individual-level risk factors may have diverse lipid profiles because of other-level risk factors resulting from different time courses, environments, cultures, policies, economies, and geographies [8,9,10]. In one multilevel study of lipid health in middle-aged Japanese workers, year-to-year changes in BMI were observed to have a considerable effect on changes in TG, HDL-C, and TC levels [33]. In a multilevel survey, conducted in 39 countries, to investigate individual and population effects on blood pressure, approximately 7–8% of individual SBP differences were attributable to population context, with population effects being particularly strong in women with a high BMI [17]. Our study revealed that school context had a significant cross-level modifying effect on the individual-level association of BMI with TG (Figure 3A and Model II in Table 4). For adolescents with BMIs of 20, 21.3 (mean), 25, 30, and 35, the VPCs were 4.9, 5.7, 8.6, 14.1, and 21.7%, respectively, resulting in a J-shaped curve (Figure 3B). This indicates that the effect of school context on individual TG levels was stronger in adolescents with high BMIs than in adolescents with low BMIs.

Epidemiological investigations have reported an association between BMI and the clustering of cardiometabolic risk factors in adolescents, with higher BMI values being linked to lower HDL-C levels [34,35]. For multilevel factors, our study also observed a 0.89 mg/dL reduction in HDL-C level for adolescents with each unit increase in BMI. Systematic reviews and meta-analyses of prospective cohort studies have suggested that a higher intake of low-fat or any dairy products was associated with a 0.72–0.79-fold decreased risk of metabolic syndrome [36]. In this study, which considered both individual-level and school-level factors, dairy intake was associated with a 0.92 higher HDL-C level, which indicates that consuming dairy products is beneficial for adolescents.

High levels of LDL-C and TC in childhood and adolescence are closely associated with adult dyslipidemia, which is a critical risk factor for cardiovascular disease [1]. Our multilevel study revealed that adolescents with an SSB intake of >500 mL/day had LDL-C and TC levels that were higher than those of non-drinkers by 5.97 and 6.12 mg/dL, respectively (Table 6, Model III). This supports the argument that high SSB consumption is a major risk factor for metabolic syndrome in adolescents [20,24,37]. Similar to our observations regarding TG levels, LDL-C and TC levels were higher in adolescents with low physical activity and increased BMI, indicating that these two individual factors have considerable effects on lipid profiles in adolescents. A physically active lifestyle during adolescence has a multitude of health benefits, including lowering the BMI [38]. Thus, promoting physical activities is a crucial strategy for the control of blood lipids in adolescents.

Schools are potentially effective venues for implementing health promotion activities. We demonstrated that students in schools with ≥2 health promotion programs per year had LDL-C levels that were 5.27 mg/dL lower than those of students in schools with 0–1 program per year. Although not all programs were designed to address healthy eating (the programs generally focused on healthy diets, weight management, oral hygiene, and smoking prevention), this result suggests that schools placing a greater emphasis on student health may provide an educational environment that aids lipid management.

In this study, students in schools with ≥46 food outlets within 600 m had TG and TC levels that were 6.90 mg/dL and 13.2–13.3 mg/dL higher, respectively, than those of students in schools with 1–45 food outlets within 600 m (Table 4 and Table 6). The high density of food outlets near schools explained 23.1 and 34.2% of the interschool variance in terms of TG and TC levels, respectively. Additionally, the high numbers of beverage shops and fast-food restaurants within 600 m of the school were the two most significant components of the association between school context and individual TG and TC levels. Adolescents are more likely to visit beverage shops and fast-food restaurants within walking distance of their school. In a large-scale longitudinal investigation of urban schoolchildren, an increase in the number of fast-food restaurants within 800 m of a school was associated with a 1.27-fold increase in the risk of obesity [39]. Although the exact mechanisms by which food outlets near schools affect blood lipids in adolescents do require further research, these data clearly highlight a correlation between the two.

Our research has several strengths. First, this is the first study using a large-scale representative sample of schools and students to analyze the effects of both individual-level and school-level factors on serum lipid profiles in adolescents. Second, the research framework and methodology of this study were designed to comprehensively assess individual-level and school-level PCV in terms of lipid levels. Third, we used VPC curves to interpret the modifying effect of school context on the individual-level association between BMI and TG level, an approach that can be used in other studies to evaluate similar data. Our research also has several limitations. First, the use of a cross-sectional design restricted all findings to the measured associations, without assessing temporality or causality. Second, the classification of health promotion programs must be refined. Because of the limited number of health programs implemented in schools, we were unable to analyze the effects of specific dietary promotion programs. Third, the present study cannot demonstrate exactly how school context affects lipid profiles in adolescents, but this question may be answered in future research.

## 5. Conclusions

Individual diet, physical activity, and BMI, as well as health promotion programs implemented in schools and the density of food outlets near schools, were observed to affect lipid profiles in adolescents in a multilevel manner. School context had a stronger cross-level effect on individual TG levels in adolescents with a high BMI than in adolescents with a normal BMI. Our findings suggest that preventive strategies to promote lipid health in adolescents should be implemented at the individual level and at the school level. In schools with a high density of nearby food outlets, adolescents with a higher BMI should receive more support from school health supervisors and intensive health education to reduce the impact of the school’s food environment on their lipid profiles, particularly their TG levels.

## Figures and Tables

**Table 1:** The distributions of lipid profiles (mg/dL) associated with demographic factors and daily calorie intake in adolescents.

Factors	Participant	Triglycerides			HDL Cholesterol			LDL Cholesterol			Total Cholesterol		
	*n* = 2727	Mean	β ^a^	*p* ^b^	Mean	β ^a^	*p* ^b^	Mean	β ^a^	*p* ^b^	Mean	β ^a^	*p* ^b^
**Categorical factors**													
**Sex**													
Boy	48.7%	75.78	Ref.		55.79	Ref.		87.98	Ref.		157.29	Ref.	
Girl	51.3%	75.44	−0.33	0.819	58.32	2.53	<0.001	91.45	3.47	0.001	164.47	7.17	<0.001
**Ethnicity**													
Fukienese	69.9%	76.55	Ref.		57.92	Ref.		90.13	Ref.		161.90	Ref.	
Hakka	10.1%	76.06	−0.49	0.846	55.81	−2.11	0.018	91.56	1.43	0.421	162.41	0.51	0.811
Mainland	10.8%	80.82	4.27	0.084	58.69	0.77	0.377	91.74	1.61	0.352	164.27	2.36	0.250
Aboriginal	9.1%	64.12	−12.43	<0.001	49.42	−8.51	<0.001	81.38	−8.75	<0.001	146.21	−15.69	<0.001
**Continuous factors**													
**Age**, year	13.43 ± 1.03		−0.13	0.857		−0.38	0.125		−0.85	0.083		−2.21	<0.001
**Daily calorie intake**, × 10^3^ kcal	2.06 ± 0.69		−0.19	0.857		0.02	0.966		−0.73	0.321		−1.34	0.126

HDL, high-density lipoprotein; LDL, low-density lipoprotein; Ref, reference. ^a^ β denotes the mean difference between reference and compared groups in categorical factors and the average change associated with a one-unit increase in continuous factors. ^b^ *p*-values for the mean differences investigated.

**Table 2:** The adjusted effects of individual-level factors on lipid profile (mg/dL) in adolescents.

Individual-Level Factors	Participant	Triglycerides			HDL Cholesterol			LDL Cholesterol			Total Cholesterol		
	*n* = 2727	aMean ^a^	adj.β ^a^	*p* ^a^	aMean ^a^	adj.β ^a^	*p* ^a^	aMean ^a^	adj.β ^a^	*p* ^a^	aMean ^a^	adj.β ^a^	*p* ^a^
**Milk intake, serving**													
<1 per week	32.4%	74.67	Ref.		56.49	Ref.		88.07	Ref.		158.77	Ref.	
≥1 per week	67.6%	76.10	1.42	0.374	57.54	1.06	0.061	90.73	2.67	0.018	162.32	3.55	0.007
**Dairy product intake, serving**													
<1 per week	54.4%	76.10	Ref.		56.18	Ref.		89.49	Ref.		160.00	Ref.	
≥1 per week	45.6%	75.17	−0.93	0.540	58.26	2.08	<0.001	90.14	0.64	0.546	162.29	2.29	0.069
**Egg intake, serving**													
<4 per week	59.4%	75.09	Ref.		56.85	Ref.		90.31	Ref.		161.11	Ref.	
≥4 per week	40.6%	76.00	0.91	0.557	57.70	0.84	0.125	89.25	−1.06	0.329	161.26	0.15	0.904
**Meat intake, serving**													
<4 per week	52.6%	75.58	Ref.		57.45	Ref.		89.47	Ref.		160.78	Ref.	
≥4 per week	47.4%	75.54	−0.04	0.978	56.69	−0.76	0.172	90.03	0.56	0.610	161.10	0.33	0.802
**Fresh fruit intake, serving**													
<1 per week	16.3%	76.27	Ref.		56.31	Ref.		88.30	Ref.		159.33	Ref.	
≥1 per week	83.7%	75.53	−0.74	0.710	57.27	0.96	0.172	90.06	1.76	0.205	161.34	2.02	0.219
**Fried food intake, serving**													
<1 per week	36.1%	74.76	Ref.		57.04	Ref.		88.80	Ref.		159.87	Ref.	
≥1 per week	63.9%	76.20	1.44	0.373	57.04	0.00	0.999	90.28	1.48	0.184	161.49	1.63	0.218
**SSB intake, mL**													
Non-intake	11.6%	74.66	Ref.		58.11	Ref.		86.80	Ref.		159.59	Ref.	
1–350 per day	39.2%	74.75	0.09	0.971	57.03	−1.07	0.205	87.47	0.67	0.688	158.80	−0.79	0.691
351–500 per day	25.5%	74.78	0.12	0.962	57.38	−0.73	0.418	91.03	4.23	0.018	162.03	2.44	0.248
>500 per day	23.7%	78.38	3.72	0.159	56.38	−1.73	0.062	93.63	6.83	< 0.001	164.11	4.52	0.037
**Physical activity, MET-min.**													
≥2140.5 per week	32.9%	72.37	Ref.		57.90	Ref.		88.03	Ref.		159.85	Ref.	
952.5–2140.5 per week	42.9%	76.80	4.43	0.010	56.87	−1.03	0.088	89.98	1.95	0.104	161.03	1.18	0.405
<952.5 per week	24.2%	77.89	5.52	0.007	56.37	−1.54	0.031	91.71	3.67	0.009	162.40	2.55	0.128
**Body mass index ^b^**	21.3 ± 4.5	-	2.66	<0.001	-	−0.84	<0.001	-	1.36	<0.001	-	0.71	<0.001

HDL, high-density lipoprotein; LDL, low-density lipoprotein; SSB, sugar-sweetened beverage; MET-min, metabolic equivalent task-minutes. ^a^ Adjusted means (aMean) and adjusted regression coefficients (adj. β) were adjusted for sex, age, ethnicity, and daily calorie intake. ^b^ Body mass index was evaluated as a continuous variable (kg/m^2^).

**Table 3:** The adjusted effects of school-level factors on lipid profile (mg/dL) in adolescents.

School-Level Factors	Participant	Triglycerides			HDL Cholesterol			LDL Cholesterol			Total Cholesterol		
	*n* = 2727	aMean ^a^	adj.β ^a^	*p* ^a^	aMean ^a^	adj.β ^a^	*p* ^a^	aMean ^a^	adj.β ^a^	*p* ^a^	aMean ^a^	adj.β ^a^	*p* ^a^
**Health promotion program in school, no.**													
0–1 per year	45.2%	76.91	Ref.		59.57	Ref.		93.06	Ref.		166.28	Ref.	
≥2 per year	54.8%	74.53	−2.39	0.108	55.04	−4.53	<0.001	87.04	−6.02	<0.001	156.60	−9.68	<0.001
**Food environment near the school**													
**Food outlet no. ^b^**													
1–45 within 600 m	49.1%	71.83	Ref.		52.01	Ref.		87.44	Ref.		153.55	Ref.	
46–140 within 600 m	20.7%	83.54	11.70	<0.001	60.55	8.54	<0.001	92.23	4.79	<0.001	168.82	15.27	<0.001
≥141 within 600 m	30.3%	76.32	4.48	0.010	62.96	10.95	<0.001	91.83	4.39	<0.001	167.66	14.10	<0.001
**Fast-food restaurant no.**													
0–1 within 600 m	77.1%	75.16	Ref.		55.62	Ref.		89.14	Ref.		159.31	Ref.	
≥2 within 600 m	22.9%	77.09	1.93	0.274	62.02	6.39	<0.001	91.84	2.70	0.027	166.56	7.25	<0.001
**Beverage shop no.**													
0–15 within 600 m	49.4%	71.90	Ref.		52.04	Ref.		87.48	Ref.		153.62	Ref.	
≥16 within 600 m	50.6%	79.22	7.31	<0.001	62.01	9.97	<0.001	91.98	4.51	<0.001	168.14	14.52	<0.001
**Eatery no.**													
0–40 within 600 m	49.1%	71.81	Ref.		52.02	Ref.		87.44	Ref.		153.55	Ref.	
≥41 within 600 m	50.9%	79.27	7.45	<0.001	61.98	9.96	<0.001	91.99	4.55	<0.001	168.13	14.58	<0.001
**Convenience store no.**													
0–5 within 600 m	37.0%	76.55	Ref.		54.30	Ref.		91.10	Ref.		160.20	Ref.	
≥6 within 600 m	63.0%	75.05	−1.50	0.328	58.73	4.43	<0.001	88.97	−2.12	0.047	161.43	1.23	0.330

HDL, high-density lipoprotein; LDL, low-density lipoprotein; no., number. ^a^ Adjusted means (aMean) and adjusted regression coefficients (adj. β) were adjusted for sex, age, ethnicity, and daily calorie intake. ^b^ Food shops, including fast-food restaurants, beverage shops, and eateries.

**Table 4:** The multilevel effects of individual and school factors on triglyceride level (mg/dL) in adolescents.

Multilevel Effects	Model I ^a^		Model II ^b^		Model III ^c^	
	Base Model		adj.β	(95% CI)	adj.β	(95% CI)
**Fixed effects**						
**Individual-level factors**						
**Physical activity, MET-min/week**						
952.5–2140.5 vs. ≥2140.5			3.72	(0.58, 6.86)	3.83	(0.69, 6.97)
<952.5 vs. ≥2140.5			5.17	(1.45, 8.90)	5.25	(1.53, 8.97)
**Body mass index, 1-unit kg/m^2^**			2.57	(2.16, 2.97)	2.59	(2.18, 2.99)
**School-level factors**						
**Health promotion program, no./year**						
≥2 vs. 0–1					−1.11	(−6.76, 4.54)
**Food outlet, no.** **within 600 m**						
≥46 vs. 1–45					6.90	(1.40, 12.39)
**Random effects**	σ^2^	(SE)	σ^2^	(SE)	σ^2^	(SE)
**Variance between schools**	77.82	(23.56)	71.55	(21.48)	59.82	(18.58)
**Variance between individuals**	1340.99	(36.57)	1188.97	(32.62)	1187.82	(32.59)
**BMI-TG slope variance between schools**			0.64 *	(0.33)	0.64 *	(0.33)
**Covariance between intercept and slope**			4.31 *	(2.04)	3.66 *	(1.92)
**PCV explained by new model** **^d^**						
Between schools	Ref.		8.1%		23.1%	
Between individuals	Ref.		11.3%		11.4%	
**Variance partition coefficient** **^d^**	0.055		0.057		0.048	

PCV, proportional change in variance; BMI, body mass index; TG, triglyceride; CI, confidence interval; σ^2^, variance; SE, standard error; no., number; * *p* < 0.05. ^a^ Model I was adjusted for sex, age, ethnicity, and daily calorie intake. ^b^ Model II represents Model I after additional adjustments for individual-level factors. ^c^ Model III represents Model II after additional adjustments for school-level factors. ^d^ The PCV and variance partition coefficient were estimated at the mean of BMI (i.e., 21.3 kg/m^2^).

**Table 5:** The multilevel effects of individual and school factors on high-density lipoprotein cholesterol level (mg/dL) in adolescents.

Multilevel Effects	Model I ^a^		Model II ^b^		Model III ^c^	
	Base Model		adj.β	(95% CI)	adj.β	(95% CI)
**Fixed effects**						
**Individual-level factors**						
**Dairy product intake, serving/week**						
≥1 vs. <1			0.94	(0.08, 1.80)	0.92	(0.06, 1.78)
**Physical activity, MET-min/week**						
952.5–2140.5 vs. 2140.5			−0.47	(−1.46, 0.52)	−0.48	(−1.47, 0.51)
<952.5 vs. ≥2140.5			−0.45	(−1.63, 0.73)	−0.47	(−1.65, 0.71)
**Body mass index, 1-unit kg/m^2^**			−0.89	(−0.98, −0.80)	−0.89	(−0.98, −0.80)
**School-level factors**						
**Health promotion program, no./year**						
≥2 vs. 0–1					−4.53	(−8.21, −0.84)
**Food outlet, no. within 600 m**						
46–140 vs. 1–45					5.98	(1.99, 9.96)
≥141 vs. 1–45					9.91	(6.01, 13.80)
**Random effects**	σ^2^	(SE)	σ^2^	(SE)	σ^2^	(SE)
**Variance between schools**	58.43	(14.36)	60.36	(14.79)	26.84	(7.03)
**Variance between individuals**	129.08	(3.52)	112.85	(3.14)	113.03	(3.15)
**PCV explained by new model**						
Between schools	Ref.		−3.3%		54.1%	
Between individuals	Ref.		12.6%		12.4%	
**Variance partition coefficient**	0.312		0.348		0.192	

PCV, proportional change in variance; CI, confidence interval; σ^2^, variance; SE, standard error; no., number. ^a^ Model I was adjusted for sex, age, ethnicity, and daily calorie intake. ^b^ Model II represents Model I after additional adjustments for individual-level factors. ^c^ Model III represents Model II after additional adjustments for school-level factors.

**Table 6:** The multilevel effects of individual and school factors on low-density lipoprotein and total cholesterol level (mg/dL) in adolescents.

	LDL Cholesterol						Total Cholesterol					
Multilevel Effects	Model I ^a^		Model II ^b^		Model III ^c^		Model I ^a^		Model II ^b^		Model III ^c^	
	Base model		adj.β	(95% CI)	adj.β	(95% CI)	Base model		adj.β	(95% CI)	adj.β	(95% CI)
**Fixed effects**												
**Individual-level factors**												
**Milk intake, serving/week**												
≥1 vs. <1			3.05	(0.97, 5.13)	2.94	(0.86, 5.02)			3.19	(0.83, 5.55)	3.13	(0.77, 5.49)
**SSB intake, mL/day**												
1–350 vs. non-intake			1.35	(−1.79, 4.49)	1.52	(−1.62, 4.66)			1.62	(−1.95, 5.18)	1.73	(−1.83, 5.29)
351–500 vs. non-intake			4.12	(0.76, 7.48)	4.25	(0.89, 7.60)			4.65	(0.84, 8.46)	4.71	(0.91, 8.52)
>500 vs. non-intake			5.87	(2.40, 9.34)	5.97	(2.50, 9.45)			6.00	(2.07, 9.94)	6.12	(2.18, 10.05)
**Physical activity, MET-min./week**												
952.5–2140.5 vs. ≥2140.5			2.24	(−0.03, 4.51)	2.23	(−0.05, 4.50)			1.90	(−0.67, 4.48)	1.90	(−0.67, 4.48)
<952.5 vs. ≥2140.5			4.15	(1.45, 6.84)	4.16	(1.47, 6.86)			3.88	(0.82, 6.94)	3.86	(0.80, 6.92)
**Body mass index, 1-unit kg/m^2^**			1.27	(1.06, 1.48)	1.27	(1.06, 1.48)			0.53	(0.29, 0.77)	0.52	(0.28, 0.76)
**School-level factors**												
**Health promotion program, no./year**												
≥2 vs. 0–1					−5.27	(−10.51, −0.02)					−8.36	(−17.46, 0.75)
**Food outlet, no.** **within 600 m**												
46–140 vs. 1–45					3.92	(−2.54, 10.37)					13.2	(3.13, 23.19)
≥141 vs. 1–45					4.23	(−1.58, 10.04)					13.3	(3.66, 23.03)
**Random effects**	σ^2^	(SE)	σ^2^	(SE)	σ^2^	(SE)	σ^2^	(SE)	σ^2^	(SE)	σ^2^	(SE)
**Variance between schools**	67.20	(18.63)	61.22	(17.18)	48.49	(14.11)	247.21	(61.97)	241.90	(60.83)	162.59	(42.10)
**Variance between individuals**	630.88	(17.21)	594.33	(16.53)	593.85	(16.52)	777.93	(21.21)	762.92	(21.21)	762.77	(21.21)
**PCV explained by new model**												
Between schools	Ref.		8.9%		27.8%		Ref.		2.1%		34.2%	
Between individuals	Ref.		5.8%		5.9%		Ref.		1.9%		1.9%	
**Variance partition coefficient**	0.096		0.093		0.075		0.241		0.241		0.176	

LDL, low-density lipoprotein; SSB, sugar-sweetened beverage; PCV proportional change in variance; CI, confidence interval; σ^2^, variance; SE, standard error; no., number. ^a^ Model I was adjusted for sex, age, ethnicity, and daily calorie intake. ^b^ Model II represents Model I after additional adjustments for individual-level factors. ^c^ Model III represents Model II after additional adjustments for school-level factors.

## Data Availability

The data are not publicly available due to privacy restrictions.

## Abbreviations

BMI: body mass index; HDL-C: high-density lipoprotein cholesterol; LDL-C: low-density lipoprotein cholesterol; MET-min: metabolic equivalent task-minutes; MLRM: multilevel linear regression models; PCV: proportional change of variance; SBP: systolic blood pressure; SSB: sugar-sweetened beverage; TC: total cholesterol; TG: triglyceride; VPC: variance partition coefficient.
